# Anti‐obesity treatment preferences of healthcare providers and people living with obesity: A survey‐based study

**DOI:** 10.1111/cob.12704

**Published:** 2024-10-28

**Authors:** Carel W. Le Roux, Anna Koroleva, Sara Larsen, Ellie Foot

**Affiliations:** ^1^ Diabetes Complications Research Centre, School of Medicine, Conway Institute of Biomolecular and Biomedical Research University College Dublin Dublin Ireland; ^2^ Market Access, Novo Nordisk Novo Nordisk A/S Søborg Denmark; ^3^ Market Access, Novo Nordisk Ipsos London UK

**Keywords:** anti‐obesity medication, obesity management, survey‐based study, treatment preference, weight loss

## Abstract

A cross‐sectional, online survey was conducted in the United Kingdom, France, Germany, and the United States (14 November–22 December 2022) to investigate preferences for anti‐obesity medication (AOM) among people with obesity (PwO) and healthcare providers (HCPs). Eligibility: Adult PwO who self‐defined their body type as overweight/obese, were trying to lose weight and had BMI ≥30.0 or 27.0–29.9 kg/m^2^ with ≥1 obesity‐related complication; HCPs had to see ≥30 PwO in a typical month and be a decision‐maker regarding their weight loss. The survey included 2500 PwO and 500 HCPs. Exercise (96%) and diet (90%) were the most common weight management methods; AOM use was low (8%). Key barriers to use of prescribed AOMs among PwO were not wanting to take AOM (34%), side effects concerns (33%), and not trusting AOM (26%). Most HCPs (79%) had prescribed/recommended AOMs. Efficacy was the most common reason for preferring one of the shown product profiles among PwO (60%) and HCPs (86%); improving cardiovascular risk was also important to 95% of HCPs when deciding which AOM to prescribe. AOM preference is largely driven by efficacy. Increasing knowledge could help to address barriers to AOM use and improve outcomes for PwO.

**What is already known about this subject?**

- Only a few small studies have investigated the perceptions and preferences of people with obesity (PwO) or their healthcare providers (HCPs) regarding the treatment of obesity and pharmacotherapy options.

**What this study adds?**

- We found that use of, satisfaction with, awareness of, and understanding of current anti‐obesity medications (AOMs) is suboptimal. Weight loss efficacy is the primary driver of AOM choice.
- We identified that the most important barriers to use of AOMs differ between HCPs and PwO, and by geographic location. To overcome these, HCPs wanted country‐specific guidelines/recommendations, whereas PwO wanted HCP recommendations, and more information on safety and oral therapies.
- There is an unmet need to improve education and awareness of AOMs for HCPs so they can treat obesity appropriately and have meaningful discussions with PwO on the full range of treatment options available. The preferences and barriers identified may also help inform the development of new AOMs in the future.

## 1 INTRODUCTION

Obesity is a serious, chronic, relapsing disease affecting 988 million adults globally in 2020 with rapidly increasing prevalence.^1^, ^2^ Guidelines recommend treatment to achieve weight loss of at least 5%–15% to reduce the risk of obesity‐related complications, such as type 2 diabetes, dyslipidaemia, cardiovascular disease, and hypertension.^3^, ^4^ Options for treatment of obesity include lifestyle modifications, pharmacotherapy, and bariatric surgery, all of which are valid options but currently have limitations. Lifestyle modifications may not lead to sufficient weight loss for the majority of patients, and many patients who have substantial weight loss regain some or all of the weight.^5^, ^6^ Pharmacological options have historically been marred by suboptimal efficacy and safety issues^7^ and, therefore, have been underutilized.^8^ However, the emergence of glucagon‐like peptide‐1 receptor agonists for treatment of obesity has provided therapeutic options that lead to clinically meaningful weight loss with improved safety and tolerability profiles.^7^, ^9^ Finally, bariatric surgery is a generally effective intervention with good safety outcomes^10^, ^11^; however, due to system capacity and resource constraints, its use tends to be limited to less than 3% of the eligible population.^12^

Current options for long‐term pharmacological treatment in the United States and Europe include orlistat,^13^, ^14^, ^15^, ^16^ naltrexone hydrochloride/bupropion hydrochloride,^17^ liraglutide 3.0 mg^18^, ^19^, ^20^ and semaglutide 2.4 mg.^21^, ^22^, ^23^ In the United States, phentermine/topiramate^24^ is also available. Each agent varies in terms of efficacy, safety/tolerability, frequency of administration (three times daily to once weekly), and route of administration (oral or subcutaneous injection). To date, there is limited evidence on preferences for treatment of obesity and perceptions of anti‐obesity medications (AOMs) among people with obesity (PwO) and healthcare providers (HCPs). A study in which preferences of PwO in Canada were investigated identified medication costs, lack of insurance coverage, and stigma as the primary barriers to PwO accessing AOMs.^25^ Nurse practitioners in the United States have also reported barriers to prescribing AOMs that were related to a lack of awareness and education.^26^ An additional study of European HCPs found that, although most participants considered obesity a disease that needs to be treated, the majority preferred to recommend lifestyle changes over AOMs.^27^ This study was performed in 2021, prior to any regulatory approvals of semaglutide 2.4 mg.

The aim of the present study was to explore the current treatment preferences and perceptions surrounding AOMs in a large population of PwO and HCPs in Europe and the United States.

## 2 MATERIALS AND METHODS

### 2.1 Study overview and participants

This was a cross‐sectional, online survey study conducted by Ipsos in the United Kingdom, France, Germany, and the United States on behalf of Novo Nordisk.

Eligible respondents were PwO aged ≥18 years who self‐defined their body type as overweight or obese, had a body mass index (BMI) (based on self‐reported height and weight) of ≥30.0 kg/m^2^ or 27.0–29.9 kg/m^2^, and had at least one obesity‐related complication. Women who were pregnant or breastfeeding, and people who self‐defined as very muscular (e.g., bodybuilders and rugby players) were excluded. Quotas for age, gender, and region of PwO were monitored in line with a representative sample. Eligible HCPs were primary care physicians (PCPs) or general practitioners (GPs), endocrinologists, and diabetologists qualified in their current specialty for 3–30 years who were seeing at least 30 PwO (BMI ≥30 kg/m^2^, or BMI 27–29.9 kg/m^2^ and obesity‐related complications) in a typical month, for whom they were one of the decision‐makers regarding their weight loss. In Germany only, gastroenterologists, bariatric surgeons, and general surgeons were also eligible if they personally performed the bariatric surgery or were involved in preparation or follow‐up; these HCPs were grouped for analysis and reported separately as ‘DE bariatric surgeons’.

### 2.2 Sample selection

The participants included in this survey study were primarily sourced from proprietary, European Society for Opinion and Marketing Research (ESOMAR)—compliant vendor panels. Survey interest was obtained via email contact, and upon confirmation of interest, invitations were sent via emails containing a unique link to the survey. Survey invitations provided only basic links and non‐leading information. IP addresses were monitored to avoid respondent duplication.

The surveys were conducted in compliance with the Market Research Society (MRS), ESOMAR, and European Pharmaceutical Market Research Association guidelines. Exemption from ethical approval was confirmed by Pearl Pathways, an independent review board. Information obtained from the surveys was handled with strict confidentiality, following the MRS Code of Conduct and all applicable laws protecting personal data and responses. Respondent data were gathered on an anonymized basis and stored for internal use only. Personal data had to be deleted after 3 months.

All participants provided written consent after being made aware of the study objectives, data confidentiality policies, and protection measures, but before starting the survey. Participation was voluntary and participants had the right to withdraw from the study at any time. Participants who completed the full survey received an honorarium.

### 2.3 Survey design

Separate surveys were developed for PwO and HCPs. Prior to launch, a qualitative phase was conducted to ensure the survey questions were framed appropriately and all potential answer options were included. The surveys were soft‐launched to allow completion of quality checks before the full survey launch. Survey completion took approximately 10 min, and participants were advised of this before starting the main survey. Adaptive questioning was used to ensure participants were only asked to respond to questions that were relevant according to their previous answers. To prevent selection bias, randomization to answer lists was performed where applicable and relevant.

PwO were asked up to 33 questions, and HCPs were asked up to 31 questions, including questions relating to opinions and preferences regarding AOMs (see Supporting Information). There was one question per page, and respondents could leave a question unanswered and come back to it but were not able to go back after a question had been answered. In the questionnaires, PwO and HCPs were shown blinded profiles of products available in their country, which were labelled A, B, X, Y, and Z for blinding purposes (Table 1). The product profiles were prepared based on approved labels of these products; the descriptions were simplified for PwO to ensure the language could be clearly understood. Product profiles A and B reflected products only available in the United States and were only shown to PwO/HCPs in the United States.

**TABLE 1:** Product profiles shown to (A) PwO and (B) HCPs.

(A)					
	A	B	X	Y	Z
Mode of action	Thought to help decrease appetite as well as reducing food cravings	Creates a long‐lasting feeling of fullness in the body after eating which reduces appetite	Controls appetite and cravings	Controls appetite and cravings	Stops fatty food from being broken down and absorbed into the body; instead, this fat is excreted
Dosing and administration	Oral tablets taken twice daily (morning and evening) but not with a high fat meal	Oral tablet taken once daily in the morning (avoid evening to prevent insomnia)	Once weekly, injection under the skin in the abdomen, thigh, or arm	Once daily, injection under the skin in the abdomen, thigh, or arm	Oral tablets taken three times daily with each main meal containing fat (during or up to 1 h after eating)
Key benefits	Average weight loss after 1 year 3.7%–8.1% reduction in bodyweight. Better control over appetite	Average 9.8%–10.9% weight loss after 1 year. Relative improvement in several risk factors associated with obesity, e.g., reduction in blood sugar levels in patients with type 2 diabetes, reduction in cholesterol levels, reduction in high blood pressure	Average weight loss after 1 year 15%. Improvement in several cardiovascular and metabolic risk factors such as high blood pressure, blood sugar, and high cholesterol levels	Average 7% weight loss after 1 year. Better control over appetite. Improvement in several cardiovascular and metabolic risk factors such as high blood pressure, blood sugar, and high cholesterol levels	Average 3% weight loss after 1 year. Improvement in several cardiovascular and metabolic risk factors such as high blood pressure and high cholesterol levels
Adverse reactions	Moderate	Severe	Moderate	Moderate	Severe

(B)					
Mode of action	Affects the hypothalamus and the mesolimbic dopamine circuit	Reduces appetite and food consumption through catecholamines released from the hypothalamus	Binds to GLP‐1 receptors to promote weight loss by regulating appetite and calorie intake	Binds to GLP‐1 receptors to promote weight loss by regulating appetite and calorie intake	Prevents enzymes from being able to hydrolyse dietary fat into absorbable fatty free acids
Dosing and administration	Oral tablets taken twice daily (morning and evening) but not with a high fat meal	Oral tablet taken once daily in the morning (avoid evening to prevent insomnia)	Once weekly, subcutaneous injection in the abdomen, thigh or upper arm	Once daily, subcutaneous injection in the abdomen, thigh or arm	Oral tablets taken three times daily with each main meal containing fat (during or up to 1 h after eating)
Key benefits	Average weight loss after 1 year 3.7%–8.1% reduction in bodyweight. Better control over appetite	Average 9.8%–10.9% weight loss after 1 year. Relative improvement in several risk factors associated with obesity, e.g., reduction in blood sugar levels in patients with type 2 diabetes, reduction in cholesterol levels, reduction in high blood pressure	Average weight loss after 68 weeks is 14.9%. Better control over appetite. Improvements in cardiovascular risk factors, e.g., blood pressure, blood sugar, cholesterol levels, HbA_1C_.	Average 7% weight loss after 56 weeks. Better control over appetite. Improvements in cardiovascular risk factors	Average 3% weight loss after 1 year. Improvement in several cardiovascular and metabolic risk factors such as high blood pressure, and high cholesterol levels
Most common adverse reactions	GI related, including nausea, diarrhoea, vomiting and constipation	Risk of addiction, itching, paraesthesia, dizziness	GI related, including nausea, diarrhoea, vomiting and constipation	GI related, including nausea, diarrhoea, vomiting and constipation	Oily spotting, flatus with discharge, faecal urgency, fatty/oily stool

Abbreviations: GI, gastrointestinal; GLP‐1, glucagon‐like peptide‐1; HbA_1C_, glycated haemoglobin; HCP, healthcare provider; PwO, people with obesity.

### 2.4 Statistical analysis

Only completed surveys were analysed, and responses from those completing the survey in under 3 min were removed if the data quality was poor or the answers did not make sense for the question.

Only fully completed surveys were included in the analysis. The results were manually analysed using quantitative analysis techniques (e.g., frequencies and mean scores). Where a rating scale was used, ‘top two box scores’ (in which the highest two responses of the scale are combined to give a single number) were analysed to summarize the data. Inferential statistical *t*‐tests or z‐tests with 95% confidence levels (*p* <.05) for each test were used to determine if there were notable differences between countries. For both surveys, total data were weighted so each country had equal proportions.

## 3 RESULTS

### 3.1 PwO survey

#### 3.1.1 Participants

A total of 2500 (France, *n* = 500; Germany, *n* = 500; United Kingdom, *n* = 500; United States, *n* = 1000) PwO participated in the survey study between 14 November and 22 December 2022. The participation rate for PwO was 87% and completion rate was 10%. Overall, the sample was 49.2% male, 49.8% female, and 0.6% non‐binary, with a mean age of 51.6 years (standard deviation [SD], 16.0), mean weight of 99.2 kg (SD, 20.6), and mean BMI of 38.2 kg/m^2^ (SD, 128.1) (Table 2A). Individual country‐level data were similar to those of the overall population, except that the mean weight of participants was higher in Germany and the United States compared with France and the United Kingdom.

**TABLE 2:** Baseline characteristics of (A) PwO and (B) HCPs.

(A)					
	United Kingdom *N* = 500	France *N* = 500	Germany *N* = 500	United States *N* = 1000	Total *N* = 2500
Sex, *n* (%)					
Male	234 (47)	246 (49)	266 (53)	489 (49)	1238 (50)
Female	260 (52)	254 (51)	230 (46)	505 (51)	1246 (50)
Non‐binary	6 (1)	0	3 (1)	4 (<1)	14 (1)
Other	0	0	1 (<1)	2 (<1)	2 (<1)
Age (years), mean (SD)	52.0 (16.6)	51.6 (16.2)	50.4 (17.1)	52.4 (13.9)	51.6 (16.0)
Weight (kg), mean (SD)	97.7 (20.5)	94.7 (17.2)	102.9 (22.0)	101.3 (21.2)	99.2 (20.6)
BMI (kg/m^2^), mean (SD)	40.1 (116.1)	43.6 (213.1)	34.4 (6.9)	34.8 (6.6)	38.2 (128.1)
Comorbidities, *n* (%)					
High blood pressure	195 (39)	166 (33)	279 (56)	505 (51)	1116 (45)
Osteoarthritis	92 (18)	130 (26)	125 (25)	167 (17)	538 (22)
Type 2 diabetes	101 (20)	87 (17)	101 (20)	196 (20)	484 (19)
Asthma	79 (16)	52 (10)	69 (14)	139 (14)	337 (13)
Too high/too low blood lipid levels	33 (7)	52 (10)	86 (17)	133 (13)	297 (12)
Obstructive sleep apnoea	34 (7)	76 (15)	42 (8)	140 (14)	278 (11)
Pre‐diabetes	34 (7)	24 (5)	12 (2)	100 (10)	150 (6)
Cardiovascular disease	26 (5)	34 (7)	20 (4)	45 (5)	128 (5)
NASH	22 (4)	7 (1)	46 (9)	41 (4)	119 (5)
Gout syndrome	18 (4)	8 (2)	34 (7)	40 (4)	100 (4)
PCOS	23 (5)	15 (3)	11 (2)	33 (3)	82 (3)
Heart failure	9 (2)	13 (3)	33 (7)	20 (2)	81 (3)
Kidney disease	14 (3)	11 (2)	28 (6)	22 (2)	80 (3)
Infertility	12 (2)	7 (1)	9 (2)	22 (2)	49 (2)
None of the above	134 (27)	126 (25)	103 (21)	212 (21)	586 (23)
Prefer not to say	3 (1)	2 (0)	1 (0)	10 (1)	14 (1)

(B)							
	France *N* = 100	Germany *N* = 150	DE non‐BS *N* = 100	DE BS *N* = 50	United Kingdom *N* = 100	United States *N* = 150	Total *N* = 500
Primary specialty, *n* (%)							
PCP/GP	50 (50)	50 (33)	50 (50)	0	50 (50)	75 (50)	229 (46)
Endocrinologist	29 (29)	11 (7)	11 (11)	0	29 (29)	75 (50)	144 (29)
Diabetologist	21 (21)	39 (26)	39 (39)	0	21 (21)	0	85 (17)
Gastroenterologist	0	31 (21)	0	31 (62)	0	0	26 (5)
Bariatric surgery specialties^a^	0	50 (33)	0	50 (100)	0	0	50 (10)
Years qualified in specialty, mean (SD)	17.4 (8.5)	17.6 (6.7)	18.7 (7.0)	15.4 (5.5)	15.0 (6.5)	15.0 (8.3)	16.2 (7.6)
Number of PwO seen in a typical month, mean (SD)	100.2 (77.6)	118.8 (105.0)	140.7 (113.2)	75.0 (68.6)	91.6 (57.0)	134.0 (95.4)	111.2 (87.1)
Responsibility for management of PwO regarding weight loss, *n* (%)							
Primary decision‐maker	77 (77)	124 (83)	91 (91)	33 (66)	77 (77)	133 (89)	407 (81)
One of the decision‐makers	23 (23)	24 (16)	9 (9)	15 (30)	23 (23)	17 (11)	92 (18)
Consulted by others	0	2 (1)	0	2 (4)	0	0	2 (0)
Affiliation with obesity centre or weight management service, *n* (%)							
Work at one	27 (27)	33 (22)	16 (16)	17 (34)	34 (34)	17 (11)	118 (24)
Formal affiliation with one	18 (18)	38 (25)	21 (21)	17 (34)	27 (27)	26 (17)	110 (22)
None	54 (54)	76 (51)	62 (62)	14 (28)	37 (37)	103 (69)	263 (53)
Do not know	1 (1)	3 (2)	1 (1)	2 (4)	2 (2)	4 (3)	10 (2)
Weight loss methods prescribed/recommended in past 6 months, *n* (%)							
Exercise	99 (99)	141 (94)	97 (97)	44 (88)	97 (97)	142 (95)	481 (96)
Diet	84 (84)	127 (85)	86 (86)	41 (82)	93 (93)	145 (97)	448 (90)
Bariatric surgery	86 (86)	111 (74)	71 (71)	40 (80)	76 (76)	127 (85)	401 (80)
Anti‐obesity medication	52 (52)	110 (73)	78 (78)	32 (64)	95 (95)	143 (95)	395 (79)
Psychological support	84 (84)	114 (76)	76 (76)	38 (76)	82 (82)	91 (61)	378 (76)
Medically supervised low‐energy diets	32 (32)	97 (65)	63 (63)	34 (68)	50 (50)	50 (33)	225 (45)
Digital applications	29 (29)	70 (47)	53 (53)	17 (34)	44 (44)	73 (49)	210 (42)
Meal replacement regimens	20 (20)	73 (49)	52 (52)	21 (42)	42 (42)	70 (47)	197 (39)
Other	0	0	0	0	2 (2)	6 (4)	8 (2)
Familiarity with anti‐obesity medications available, *n* (%)							
Very familiar	23 (23)	54 (36)	35 (35)	19 (38)	45 (45)	84 (56)	200 (40)
Fairly familiar	46 (46)	74 (49)	52 (52)	22 (44)	50 (50)	60 (40)	232 (46)
Not very familiar	26 (26)	21 (14)	12 (12)	9 (18)	5 (5)	6 (4)	61 (12)
Not at all familiar	5 (5)	1 (1)	1 (1)	0	0	0	7 (1)

Abbreviations: BMI, body mass index; BS, bariatric surgeon; DE, Germany; GP, general practitioner; HCP, healthcare provider; NASH, non‐alcoholic fatty liver disease; PCOS, polycystic ovary syndrome; PCP, primary care physician; PwO, people with obesity; SD, standard deviation.

^a^ Includes gastroenterologists, bariatric surgeons, general surgeons, and gastroenterology surgeons.

#### 3.1.2 Product profile preference

**FIGURE 1:**
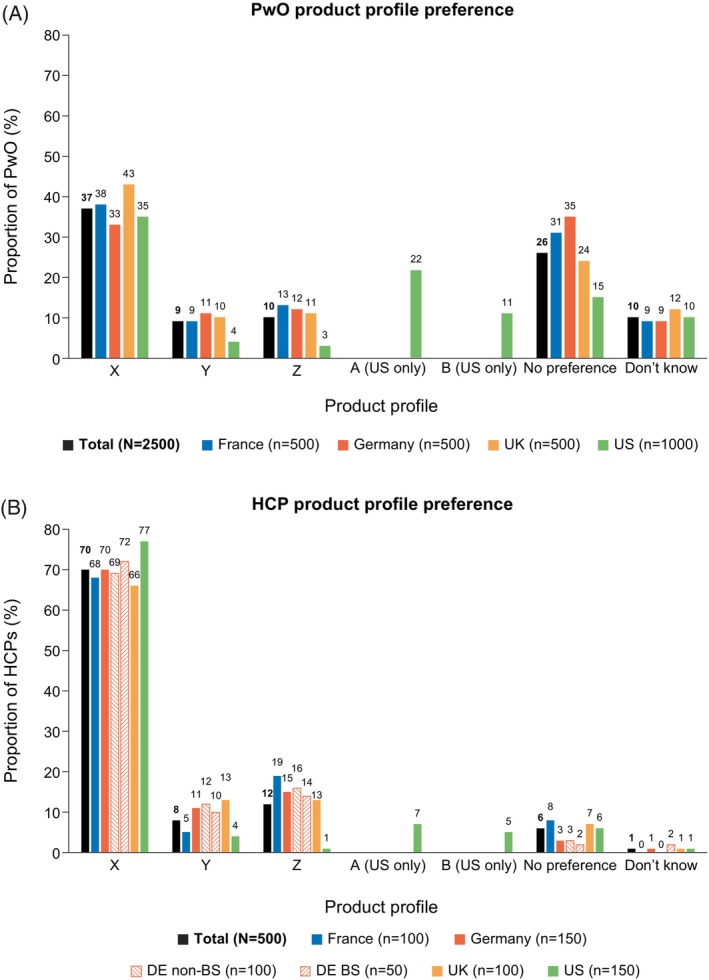
Product profile preference among (A) PwO and (B) HCPs. BS, bariatric surgeon; DE, Germany; HCP, healthcare provider; PwO, people with obesity.

Based on the profiles shown, product X was preferred by the highest proportion of surveyed PwO (37%; Figure 1A), including in the United States, where PwO also had the option to select preference for products A and B. ‘No preference’ was selected by 26% of PwO. The three most common reasons for preference of product X were effective weight loss (60%), less side effects (45%), and how often it is taken (43%) (Table 3A). The top three factors that would make PwO consider using product X were more detail on side effects, especially for PwO in the United States (overall, 44%; France, 38%; Germany, 43%; United Kingdom, 44%; United States, 52%), an HCP recommendation (overall, 41%; France, 48%; Germany, 31%; United Kingdom, 41%; United States, 42%) and a daily tablet instead of an injection (overall, 36%; France, 34%; Germany, 34%; United Kingdom, 36%; United States, 41%).

**TABLE 3:** Reasons for preference of product profile X among (A) PwO and (B) HCPs.

(A)					
	United Kingdom *N* = 500	France *N* = 500	Germany *N* = 500	United States *N* = 1000	Total *N* = 2500
*n*	214	189	163	351	927
Reason, *n* (%)					
Effective weight loss	132 (62)	111 (59)	74 (45)	256 (73)	556 (60)
Less side effects	108 (50)	91 (48)	70 (43)	136 (39)	421 (45)
How often it is taken	107 (50)	58 (31)	74 (45)	158 (45)	398 (43)
More tolerable side effects	75 (35)	87 (46)	47 (29)	146 (42)	352 (38)
Additional health benefits	65 (30)	63 (33)	38 (23)	121 (34)	283 (31)
How it is taken (injection/oral pill)	50 (23)	50 (26)	41 (25)	76 (22)	224 (24)
How it works	47 (22)	39 (21)	32 (20)	84 (24)	200 (22)
Another benefit	4 (2)	2 (1)	4 (2)	6 (2)	16 (2)
Do not know	2 (1)	1 (1)	2 (1)	0	6 (1)

(B)							
	France *N* = 100	Germany *N* = 150	DE non‐BS *N* = 100	DE BS *N* = 50	United Kingdom *N* = 100	United States *N* = 150	Total *N* = 500
*n*	68	105	69	36	66	115	351
Reason, *n* (%)							
Weight loss efficacy	61 (90)	83 (79)	54 (78)	29 (81)	56 (85)	104 (90)	302 (86)
Frequency of administration	35 (51)	48 (46)	34 (49)	14 (39)	32 (48)	50 (43)	165 (47)
Additional benefits	11 (16)	41 (39)	32 (46)	9 (25)	17 (26)	41 (36)	103 (29)
Mode of action	24 (35)	40 (38)	24 (35)	16 (44)	9 (14)	35 (30)	104 (30)
Dosing	13 (19)	36 (34)	22 (32)	14 (39)	20 (30)	22 (19)	90 (26)
Safety profile	7 (10)	30 (29)	20 (29)	10 (28)	8 (12)	44 (38)	80 (23)
Method of administration	11 (16)	29 (28)	19 (28)	10 (28)	7 (11)	15 (13)	59 (17)
Indication	2 (3)	20 (19)	15 (22)	5 (14)	5 (8)	12 (10)	35 (10)
Another benefit	0	2 (2)	1 (1)	1 (3)	1 (2)	3 (3)	5 (2)
Do not know	0	1 (1)	0	1 (3)	0	0	1 (0)

Abbreviations: BS, bariatric surgeon; DE, Germany; HCP, healthcare provider; PwO, people with obesity.

#### 3.1.3 Current methods of weight management, treatment preferences and satisfaction

The three most common current methods of weight management were healthy eating (73%), increased exercise (58%), and calorie counting (33%) (Figure S1A). Approximately two‐thirds of surveyed PwO currently using healthy eating or increased exercise as a weight loss approach were satisfied with these methods (healthy eating, *n* = 1187/1823, 65%; increased exercise, *n* = 921/1442, 64%), whereas a slightly lower proportion was satisfied with medically supervised low‐energy diets (*n* = 48/84, 57%) and calorie counting (*n* = 427/832, 51%). Satisfaction with digital apps (*n* = 182/298, 61%) and bariatric surgery (*n* = 53/74, 72%) was also high.

Current use of weight loss medication was very low among the surveyed PwO overall (8%), and satisfaction among those currently using this method varied between countries (overall, 96/190, 51%; France, *n* = 11/30, 37%; Germany, *n* = 14/42, 33%; United Kingdom, *n* = 31/46, 67%; United States, *n* = 41/68, 60%). Only 3% of surveyed PwO (*n* = 74) had received bariatric surgery.

**FIGURE 2:**
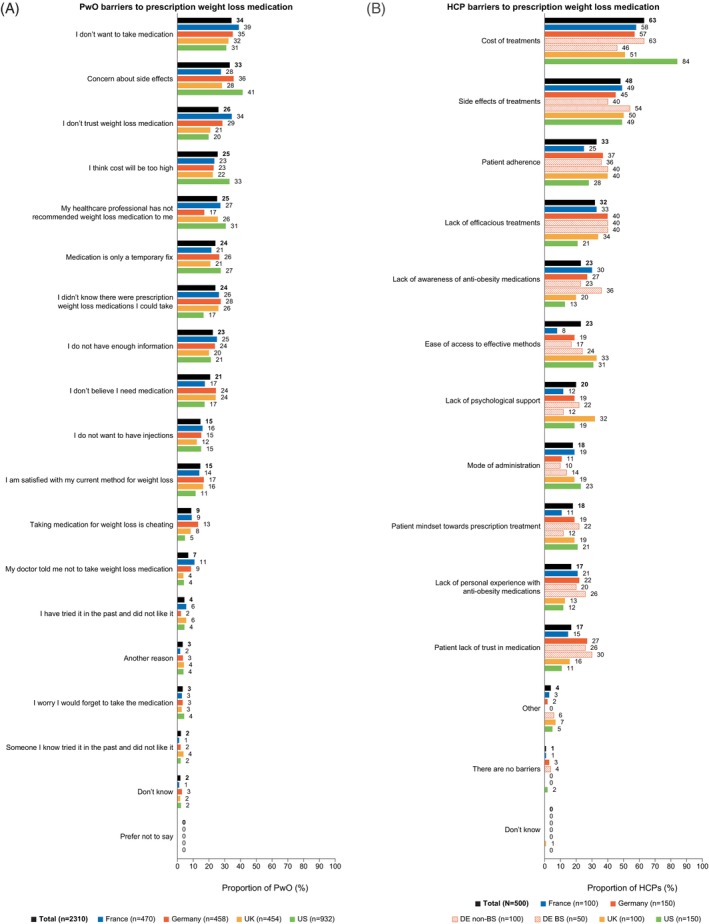
Barriers to (A) using prescription weight loss medication among PwO and (B) prescribing weight loss medication among HCPs. BS, bariatric surgeon; DE, Germany; HCP, healthcare provider; PwO, people with obesity.

Of those not using a prescription weight loss medication (*n* = 2310), the three most common reasons for this were not wanting to take medication (34%), concerns about side effects (33%) and not trusting the medication (26%) (Figure 2A). The proportion of PwO concerned about side effects was higher in Germany and the United States compared with in France and the United Kingdom (36% and 41% vs. 28% and 28%, respectively), and the proportion of PwO in France and Germany who did not trust weight loss medication was higher than in the United Kingdom and the United States (34% and 29% vs. 21% and 20%, respectively). Notably, the proportion of surveyed PwO who thought the cost would be too high was higher in the United States (33%) than in France, Germany, and the United Kingdom (range, 22%–23%). Almost one‐quarter of PwO overall (24%) reported that they did not know there were prescription weight loss medications available that they could take, with higher proportions reporting this in European countries (26%–28%) compared with the United States (17%). Additionally, one in five (21%) PwO did not believe they needed medication.

### 3.2 HCP survey

#### 3.2.1 Participants

A total of 500 HCPs (France, *n* = 100; Germany, *n* = 150; United Kingdom, *n* = 100; United States, *n* = 150) took part in the survey study between 14 November and 13 December 2022. HCP participation rate was 96% and completion rate was 56%. HCPs were recruited according to quotas; 46% were PCPs or GPs, 29% were endocrinologists and 17% were diabetologists (Table 2B). Germany had a lower proportion of endocrinologists (7%) and a higher proportion of diabetologists (26%) than other countries and also included 50 bariatric surgeons. HCPs had been qualified in their specialty for a mean of 16.2 years (SD, 7.6) and saw a mean of 111.2 (SD, 87.1) PwO in a typical month, although this ranged from 75.0 per month for DE bariatric surgeons to 140.7 per month for DE non‐bariatric surgeons.

#### 3.2.2 Product profile preference

The majority of surveyed HCPs (70%) would prefer to prescribe product X to PwO (Figure 1B). Preference for product X was higher among US HCPs (77%) compared with those in European countries (66%–70%). Among those who would prefer to use product X (*n* = 351), the three most common reasons for this preference were weight loss efficacy (86%), frequency of administration (47%), and mode of action (30%) (Table 3B). Of all surveyed HCPs, 62% believed that the PwO they treat would prefer product X.

To encourage recommendation of product X to PwO, 67% of surveyed HCPs in European countries (*n* = 375) wanted guidelines from health organizations or medical societies in their country (France, 57%; Germany, 68% [non‐bariatric surgeons, 68%; bariatric surgeons, 68%]; Unite Kingdom, 76%), 56% wanted a recommendation from a health authority/health technology assessment body in their country (France, 56%; Germany, 51% [non‐bariatric surgeons, 50%; bariatric surgeons, 52%]; United Kingdom, 62%), 42% wanted to know whether the PwO had responded to previous medications (France, 35%; Germany, 52% [non‐bariatric surgeons, 59%; bariatric surgeons, 38%]; United Kingdom, 38%) and 34% wanted more information in general (France, 21%; Germany, 33% [non‐bariatric surgeons, 31%; bariatric surgeons, 38%]; United Kingdom, 47%). A small proportion (≤30%) of HCPs would be encouraged to recommend product X by patient requests, peer recommendations, or visits from sales representatives.

#### 3.2.3 Current prescribing behaviours and satisfaction with currently available AOMs

The weight loss interventions most commonly prescribed or recommended by surveyed HCPs in the past 6 months were exercise (96%) and diet (90%), as shown in Figure S1B. The majority of surveyed HCPs (79%) had also prescribed or recommended AOMs, with more HCPs prescribing or recommending AOMs in the United Kingdom and the United States (both 95%) compared with France (52%) and Germany (73% [non‐bariatric surgeons, 78%; bariatric surgeons, 64%]).

Around half (48%) of the surveyed HCPs were satisfied with the AOMs available in their country, with lower rates of satisfaction in Europe (France, 30%; Germany, 39%; United Kingdom, 58%) compared with the United States (64%), where additional AOMs, corresponding to product profiles A and B, are available. The three most common barriers to prescribing AOMs were cost (63%), side effects (48%), and patient adherence (33%) (Figure 2B). Cost was more likely to be a barrier to prescribing in the United States (84%) than in European countries (United Kingdom, 51%; France, 58%; Germany, 57% [non‐bariatric surgeons, 63%; bariatric surgeons, 46%]). When asked what the most important outcomes for AOMs were, the three most common answers among the surveyed HCPs were sustained weight loss (78%), reduction of comorbidities (67%), and improved quality of life (63%). Also, when asked, 95% of surveyed HCPs felt that improvement of cardiovascular risk factors was important when prescribing AOMs.

## 4 DISCUSSION

This cross‐sectional, survey‐based study of PwO and HCP treatment preferences provides new insights into preferences and barriers related to pharmacological management of obesity. Among those surveyed, lifestyle modifications were favoured for weight management, AOMs were under‐utilized and only around half of surveyed PwO and HCPs were satisfied with current AOMs. Key barriers to use of AOMs were identified, and we observed important differences between HCPs and PwO as well as geographical differences. Notably, more PwO in France and Germany did not trust AOMs compared with PwO in the United Kingdom and the United States, where AOMs are more commonly prescribed. Taken together, these findings indicate there is an unmet need to develop better AOMs; the barriers and priorities identified in this study may help inform this process.

The proportion of surveyed PwO with a product profile preference was smaller than that of HCPs; however, in both groups, there was a preference for product profile X (PwO, 37%; HCPs, 70%). The main driver of this preference was weight loss efficacy (PwO: 60%; HCPs: 86%). Reasons for the preference of product X among surveyed PwO included side effects (45%), tolerability (38%) and frequency of administration (43%); only 23% of surveyed HCPs indicated the safety profile as a reason for preferring product X. A portion of this difference may be attributable to the fact that only top‐line information on the product safety profile (i.e., moderate/severe) was provided to PwO in the product profiles. Other potential explanations include the impact that side effects could have on quality of life, lack of information about long‐term side effects and previous negative experiences with AOMs.^25^ In contrast, surveyed HCPs are likely to be more familiar with, and feel more comfortable in their understanding of, safety data than PwO. This observation demonstrates a need for HCPs to proactively discuss safety concerns and safety data with PwO and to provide reassurance.

Complications of obesity are a major cause of morbidity and mortality among PwO, with cardiovascular disease and diabetes being the leading causes of death related to high BMI.^1^ The product profiles provided to PwO and HCPs in the study also outlined key benefits beyond weight loss alone—e.g., reductions in blood sugar levels associated with type 2 diabetes and improvements in cardiovascular risk factors. PwO has previously acknowledged additional health benefits as a desirable outcome,^25^ and approximately two‐thirds of HCPs are reported to have prescribed a weight loss treatment to reduce the burden of an obesity‐related complication.^27^ Moreover, we found that the majority of surveyed HCPs felt that cardiovascular improvements were important when prescribing AOMs, but only one‐third of surveyed PwO indicated additional health benefits as a reason for preferring product X. This indicates that education is needed to inform PwO on the importance of managing obesity‐related complications and that AOMs can offer benefits in addition to weight loss, especially cardiovascular benefits. Emerging data will help further understand obesity and inform treatment decisions that take into consideration not only obesity but also its potential complications.

Diet and exercise are the cornerstone of weight management and are recommended alongside all other obesity treatments, including AOMs. Therefore, as expected, diet and exercise were the most common weight loss interventions prescribed or recommended by surveyed HCPs in the past 6 months. However, it is well documented that use of these methods alone tends to be unsuccessful for the majority of PwO to achieve and sustain long‐term weight loss.^28^ Over three‐quarters (79%) of surveyed HCPs have prescribed or recommended AOMs in the past 6 months, yet only 8% of PwO were currently receiving AOMs. This paradoxical finding could be attributable to the survey response including a ‘recommended’ option, which would allow HCPs to select this response for PwO not prescribed AOM. It is also possible that PwO may not be filling prescriptions or adhering to medications.^25^

The proportion of HCPs prescribing/recommending AOMs and their satisfaction with AOMs was higher in the United Kingdom and United States compared with France and Germany. This may be explained, at least in part, by the availability of semaglutide 2.4 mg in the United States but not Europe when the study was conducted. Differences could also be due to increased public awareness, particularly in the United Kingdom. This survey was carried out in November and December 2022, during which time the National Institute for Health and Care Excellence was appraising semaglutide 2.4 mg (final guidance published March 2023)^29^ leading to substantial press coverage in the United Kingdom. Despite higher use of AOMs in the United States, more HCPs and PwO cited cost as a barrier to use compared with the European countries. This is likely a reflection of differences in healthcare system infrastructure and insurance coverage^30^, ^31^ and is aligned with findings from the Canadian study, which identified treatment cost and lack of insurance coverage as major barriers to AOM access.^25^

A wide range of barriers to use of AOMs were explored in this study. The barriers identified by surveyed PwO overlapped with those identified in the Canadian study^25^ and a United States survey of nurse practitioners,^26^ including costs, side effects, route of administration, lack of awareness, and stigma surrounding obesity and the use of pharmacotherapy for its treatment. Previous studies identified an HCP knowledge gap regarding methods to treat obesity and the availability of AOMs.^26^, ^27^ Our study confirms this, with 23% of surveyed HCPs citing lack of awareness of AOMs as a barrier to prescribing. Given the wide, heterogeneous range of barriers to use of AOMs, a range of treatment options for weight management would be beneficial for PwO and HCPs.

There now appears to be increased awareness and acceptance of obesity as a disease among HCPs,^27^ but our study did not explore whether HCPs differentiate between treatments for obesity and treatments for weight loss; these concepts are often thought of interchangeably. Moreover, many PwO surveyed in this study felt they should be able to lose weight without medication. This may partly relate to PwO also not distinguishing between treatments for weight loss and those for obesity. We hypothesize that a greater focus on the need to treat the disease of obesity would prompt HCPs to seek education and proactively discuss all treatment options with PwO, empowering PwO to make informed decisions about their disease. The insights gained from this study regarding the information, guidance and drug characteristics that PwO and HCPs wanted in order to consider use or recommendation of product X are informative for enabling this approach. Specifically, surveyed PwO wanted more information about side effects and a recommendation from an HCP, suggesting that HCP communication and confidence is important for the reassurance of PwO. Surveyed HCPs (in Europe) indicated that they want country‐level guidance/recommendations from professional organizations and health authorities. Given the public health implications of obesity,^32^ ensuring that local guidelines and recommendations are kept up to date should be a priority for governments and health authorities. In turn, this may give HCPs more confidence to proactively discuss the availability of AOMs and make recommendations to PwO.

We also note that among surveyed PwO, 36% indicated that a daily pill instead of a weekly injection would make them consider product X, highlighting a gap for an oral AOM with a similar profile to product X. In the meantime, barriers relating to the route of administration could be overcome, at least in some cases, with education or product demonstrations, as a previous study found that injectable administration was more acceptable to those who had (vs. had not) experience using injectables, and seeing the needle size also helped address fear of injection.^25^

A key strength of this study is that it was, to the best of our knowledge, the first multinational study investigating preferences of both PwO and HCPs regarding AOMs in Europe and the United States. The study also included higher participant numbers than previous studies.^25^, ^26^, ^27^ Randomization of answer options helped prevent selection bias, and the online surveys in this study were conducted in a local language and allowed participants across different demographics to be included, to ensure a good spread in each country. In addition, all HCP participants were decision‐makers regarding treatment for weight loss, so were knowledgeable and experienced in the area. While these features help increase the generalizability of our findings, it should be noted that the survey sample is not representative of the general population of PwO or HCPs. The survey was only performed in four countries, and there were potential sources of recruitment bias, including use of email for recruitment, and having PwO self‐define as being obese or overweight and self‐declare that they were trying to lose weight. This sample also does not include institutionalized PwO or those with the most severe complications and disabilities. A further limitation is that the online survey design provides the potential for inaccurate recall and false reporting, and some participants did not fully complete the survey and, therefore, were not included in analyses. Considering the large sample size and the omission of partially completed questionnaires from the final data set, this limitation is not expected to bias the survey outcomes.

## 5 CONCLUSION

We observed that a weight loss efficacy of 15% drove AOM preference among PwO and HCPs; cardiovascular improvement was also an important factor for 95% of HCPs. PwO show more hesitancy regarding AOMs than HCPs, with side effects and tolerability being major concerns for PwO. There is a need for HCPs to treat obesity in the same way as other chronic diseases, to increase their knowledge and awareness of AOMs, and to improve communication with PwO regarding pharmacological options to increase uptake of AOMs in PwO who may benefit from such treatment. There is also a need for governments and health authorities to provide HCPs with guidance on use of AOMs and for a greater therapeutic armamentarium of AOMs that addresses current barriers to their use.

## AUTHOR CONTRIBUTIONS

CWLR contributed to writing and editing of the manuscript. AK provided medical input from Novo Nordisk and reviewed materials. SL was the Novo Nordisk project lead and reviewed all documents related to the study. EF was part of the team that collected and analysed the data.

## FUNDING INFORMATION

This study was sponsored by Novo Nordisk A/S.

## CONFLICT OF INTEREST STATEMENT

CWLR reports grants from AnaBio, the Health Research Board, the Irish Research Council, and Science Foundation Ireland. He serves on advisory boards and speaker panels for Boehringer Ingelheim, Currax, Eli Lilly, G.I. Dynamics, Glia, Herbalife, Irish Life Health, Johnson & Johnson, Novo Nordisk, Rhythm Pharmaceuticals, and Zealand Pharma. CWLR is a member of the Irish Society for Clinical Nutrition and Metabolism outside the area of work commented on here; he was the chief medical officer and director of the Medical Device Division of Keyron in 2021. Both of these are unremunerated positions. CWLR was a previous investor in Keyron, which develops endoscopically implantable medical devices intended to mimic the surgical procedures of sleeve gastrectomy and gastric bypass. No survey participants have been included in any of Keyron's studies and Keyron is not listed on the stock market. CWLR was gifted stock holdings in September 2021 and divested all stock holdings in Keyron in September 2021. He continues to provide scientific advice to Keyron for no remuneration. CWLR provides obesity clinical care in the Beyond BMI clinic and is a shareholder. AK and SL are Novo Nordisk employees and shareholders. EF is an employee of Ipsos, which was commissioned to conduct this research.

## Supporting information


**Data S1** Supporting Information

## Data Availability

The datasets generated during and/or analysed during the current study are available from the corresponding author on reasonable request.
