# Supplementary material for: Anti‐obesity treatment preferences of healthcare providers and people living with obesity: A survey‐based study
Source: Clin Obes. 2024 Oct 28;15(1):e12704. doi: 10.1111/cob.12704 (PMC11706755; doi:10.1111/cob.12704)
Supplement: Supplementary file 1 — Data S1 Supporting Information [file COB-15-e12704-s001.pdf]

Anti-obesity treatment preferences of healthcare providers and people living with obesity: A survey-based study

Carel W Le Roux,[1] Anna Koroleva,[2] Sara Larsen,[2] Ellie Foot[3]

[1]Diabetes Complications Research Centre, School of Medicine, Conway Institute of Biomolecular and Biomedical Research, University College Dublin, Dublin, Ireland; [2]Novo Nordisk A/S, Søborg, Denmark; [3]Ipsos, London, UK

Correspondence

Sara Larsen, Novo Nordisk A/S, Vandtårnsvej 108, 2860 Søborg, Denmark

xsla@novonordisk.com

Supporting information

PwO survey

Screening questions

Response options are shown in the bulleted list beneath each question. No other response options were available.

In which country do you live?
- United Kingdom
- France
- Germany
- United States

Which of the following describes how you think of yourself?
- Male
- Female
- Non-binary
- In another way

Please record your age in the space provided, as of your last birthday:
- Free text

Region

United Kingdom
- Northeast and Yorkshire
- Northwest
- Midlands
- Southwest and Wales
- East and Southeast
- London
- Scotland
- Northern Ireland

France
- Ille
- Nord est
- Nord oest
- Oest
- Sudeast

Germany
- North
- East
- West
- South

USA

- Northeast
- Midwest
- West
- South

Please record your height in the space provided (cm):
- Free text

Please record your weight in the space provided (kg):
- Free text

Calculated BMI
- Autogenerated

Which of the following health conditions, if any, have you been personally diagnosed with by a healthcare professional (e.g. doctor or nurse)?
- High blood pressure
- Osteoarthritis
- Type 2 diabetes
- Asthma
- Too high or too low blood lipid (fat) levels
- Obstructive sleep apnoea
- Pre-diabetes
- Cardiovascular disease
- Non-alcoholic fatty liver disease (NASH)
- Gout syndrome
- Polycystic ovary syndrome (PCOS)
- Heart failure
- Kidney disease
- Infertility
- None of the above

How would you best describe your current body type?
- Overweight
- Obese

Main questionnaire

SECTION A: TREATMENT JOURNEY

A1. Firstly, which of the following, if any, are the **main reasons** you are currently trying to lose weight?
- To make myself feel better
- To reduce waist size
- To feel more confident in myself
- Help with other conditions
- Recommendation from healthcare professional, e.g., GP, nurse, dietician, physiotherapist

- Prevent or reverse diabetes
- Friend/family recommendation
- Another reason
- None of the above
- Prefer not to say

A2. Which of the following, if any, do you believe are the **challenges** you currently face when trying to lose weight?

- Lack of motivation
- Cannot find a method which is easy for me to follow
- Lack of effective weight loss methods
- High costs
- Lack of knowledge on the different weight loss methods
- Stigma around people overweight/ living with obesity losing weight
- Lack of medical support
- Healthcare professionals reluctant to prescribe weight loss medication
- Lack of support from friends/ family
- Healthcare professionals not listening to my weight loss needs
- Healthcare professionals reluctant to discuss treatment options
- Other challenge
- None of the above
- Don’t know

A3. You mentioned you are currently trying to lose weight, which weight loss methods are you **currently** using?

- Healthy eating
- Increased exercise
- Calorie counting
- Weight loss programmes
- Digital applications (apps)
- Meal replacement regimens
- Weight loss medication
- Medically supervised low energy diets
- Bariatric surgery
- Other
- I am not currently using anything to lose weight
- Don’t know

A4. Thinking of the medication that you are currently taking to lose weight; is it pills taken orally or injection or both pills and injection together?

- Pills taken orally only
- Pills and injections together
- Injections only
- Don’t know

A5. To what extent are you satisfied with your current method for weight loss, if at all?

- Very satisfied
- Fairly satisfied
- Not very satisfied

- Not at all satisfied
- Don’t know

A6. How involved are you in making decisions about your weight loss methods, if at all?
- Very involved
- Fairly involved
- Not very involved
- Not at all involved
- Don’t know

A7. What are the main factors you take into consideration when choosing which weight loss method to use?
- Effective weight loss
- Continued weight loss
- Easy to use
- Control of hunger
- Impact on quality of life
- Low cost
- Convenience
- Lack of side effects
- Not wanting to take a drug
- Minimal effort needed
- Fast weight loss
- No need to speak to a doctor
- Healthcare professional recommendation
- Friend or family recommendation
- How the drug is administered
- Another factor
- Don’t know
- Prefer not to say

A8. What is your main reason(s), if any, for not using prescribed weight loss medication?
- I don’t want to take medication
- Concern about side effects
- I don’t trust weight loss medication
- I think cost will be too high
- My healthcare professional has not recommended weight loss medication to me
- I didn’t know there were prescription weight loss medications I could take
- Medication is only a temporary fix
- I do not have enough information
- I don’t believe I need medication
- I do not want to have injections
- I am satisfied with my current method for weight loss
- Taking medication for weight loss is cheating
- My doctor told me not to take weight loss medication
- I have tried it in the past and did not like it
- I worry I would forget to take the medication
- Someone I know tried it in the past and did not like it

- Another reason
- Don’t know
- Prefer not to say

A9. Thinking of the last 2 years, how many different methods have you used to lose weight?
- Free text box

A10. To what extent do you agree or disagree with the following statements:

Obesity is a condition that requires lifelong treatment

Currently available weight loss methods are not a long-term solution

- Strongly agree
- Slightly agree
- Neither agree nor disagree
- Slightly disagree
- Strongly disagree
- Don’t know

SECTION B: PROFILE REVIEW

B1. What are your initial reactions, if any, to product X?
- Very positive
- Fairly positive
- Neither positive nor negative
- Fairly negative
- Very negative
- Don’t know

B2. How likely, or unlikely, would you be to use product X to help reduce your weight if recommended by your doctor?
- Very likely
- Fairly likely
- Neither likely nor unlikely
- Not very likely
- Not at all likely
- Don’t know

B3. What do you believe are the **main benefits**, if any, of product X?
- Regulates appetite
- Only needs to be taken once a week
- Effective weight loss
- Improvements in cardiovascular risk factors
- Convenience
- Regulates food intake
- Tolerable side effects

- Injectable
- Another benefit
- No benefit
- Don’t know

B4. What would be your **main concerns**, if any, of using product X?
- Severity of side effects
- Long term effect
- Injection
- The way it works in the body
- Whether it’s suitable for me
- How long I would have to take it for
- How long it takes to be fully effective
- How often it has to be taken
- Another concern
- No concerns
- Don’t know

B5. What, if anything, would make you consider using product X to help reduce your weight?
- More detail on side effects
- Healthcare professional recommendation
- Daily pill instead of injection
- More information on product X
- Patient testimonies from those who have used product X
- Cheaper than other weight loss methods
- Having a healthcare professional administer the injection
- Something else
- Nothing, I already feel comfortable
- Don’t know

B6. Which, if any, of the products you have just seen would you prefer to use?
- Product X
- Product Z
- Product Y
- Product A (US only)
- Product B (US only)
- No preference
- Don’t know

B7. You previously stated you would prefer to use **[product profile selected from B6]**, why is that?
- Effective weight loss
- Less side effects
- How often it’s taken
- More tolerable side effects
- Additional health benefits
- How it’s taken (injection/ oral pill)
- How it works

- Another benefit
- Don’t know

SECTION C: TYPING TOOL

C1. How much do you agree or disagree with the statements listed below?

When I look at the story of my life, I am pleased with how things have turned out so far

I gave up trying to make big improvements or changes in life a long time ago

Regardless of my weight, I feel that I am just as competent as anyone

Because of my weight I don’t feel like my true self

- Strongly agree
- Somewhat agree
- Neither agree nor disagree
- Somewhat disagree
- Strongly disagree

C2. Use the marker to indicate how much you feel your overall health is under your personal control

- Marker scale: 0 = not at all under my control and 100 = completely under my control

C3. How much, if at all, do the following statements reflect your opinion about anti-obesity medications?

I should be able to lose weight without medication

My doctor doesn’t know enough about anti-obesity medications to make me feel confident in their effectiveness

- Completely reflects my opinion
- Mostly reflects my opinion
- Somewhat reflects my opinion
- Slightly reflects my opinion
- Does not reflect my opinion at all

C4. If you started an anti-obesity medication, how quickly would you need to see results?

- Less than 1 month
- 1 month to less than 3 months
- 3 months to less than 6 months
- 6 months to less than 1 year
- 1 year or more
- I am not concerned with how quickly I see results as long as there is a long term benefit

HCP survey

Screening questions

Response options are shown in the bulleted list beneath each question. No other response options were available.

In which country do you live?

- United Kingdom
- France
- Germany
- United States

Which of the following best describes your primary medical specialty?

- PCP/GP
- Endocrinologist
- Diabetologist
- Bariatric surgery specialities
    - Gastroenterologist
    - Bariatric surgeon
    - General surgeons
    - Gastro surgeons
- None of the above
- Prefer not to say

For approximately how many years have you been qualified in your current primary medical specialty?

- Free text

How many people living with obesity or overweight do you see in a typical month for weight-related discussions, if any?

- Free text

Which of the following, if any, best describes your responsibility for the management of people living with obesity or overweight, regarding their weight loss decisions?

- I am the primary decision maker
- I am one of the decision makers
- I am consulted on my opinion by others making the final decision

Which of the following, if any, best describes your involvement in bariatric surgery? (this question only applied to Germany respondents involved in bariatric surgery):

- I personally perform bariatric surgery
- I am involved in preparation and/or follow up for bariatric surgery, but do not personally perform the surgery
- I am not involved in the bariatric surgery process
- I do not know

Do you personally work within or have a formal affiliation with an obesity centre/a specialist weight management service?

- I work at one of these centres
- I have a formal affiliation with one of these centres
- I do not work at and do not have a formal affiliation with one of the centres
- Don’t know

Main questionnaire

SECTION A: CURRENT OBESITY LANDSCAPE

A1. Firstly, how involved are patients in making decisions about their weight loss methods if at all?
- Very involved
- Fairly involved
- Not very involved
- Not at all involved
- Don’t know

A2. What weight loss methods, if any, have you prescribed or recommended for the treatment of obesity in the past 6 months?
- Exercise
- Diet
- Bariatric surgery
- Anti-obesity medication
- Psychological support
- Medically supervised low energy diets
- Digital applications
- Meal replacement regimens
- Another weight loss method
- I have not prescribed or recommended any methods in the past 6 months
- Don’t know

A3A. Overall, how familiar are you with the anti-obesity medications currently available in your market, if at all?
- Very familiar
- Fairly familiar
- Not very familiar
- Not at all familiar
- I do not know

A3B. How satisfied or dissatisfied are you with the anti-obesity medication currently available in your market?
- Very satisfied
- Fairly satisfied
- Neither satisfied nor dissatisfied
- Fairly dissatisfied
- Very dissatisfied
- Don’t know

A4. Which of the following, if any, best describes the most important outcomes you want an anti-obesity medication to achieve?

- Sustained weight loss
- Reduction of comorbidities
- Improved quality of life
- Improved mobility
- Appetite suppression
- Weight loss in a certain time
- None of the above
- Don’t know

A5. What are the main patient characteristics that would influence you to prescribe currently available anti-obesity medications, if any?

- Comorbidities
- Failed on diet and exercise alone
- BMI meets the criteria of anti-obesity medication label
- BMI of at least 30
- Patient mindset
- Requesting anti-obesity medication
- Treatment history
- Preparation for bariatric surgery
- Mental health
- Age
- Gender
- Ethnicity
- Other
- No patient characteristic would influence me to prescribe anti-obesity medications
- Don’t know

A6. What are the main TREATMENT characteristics that would influence you to prescribe currently available anti-obesity medications, if any?

- Treatment efficacy
- Treatment safety
- Cost of treatment
- Additional comorbidities treated
- Supported in guidelines
- Guidelines
- Mode of action
- Method of administration
- Duration of treatment
- Frequency of administration
- Other
- No treatment characteristic would influence me to prescribe anti-obesity medications
- Don’t know

A6B. To what extent, is an improvement on cardiovascular risk factors important to you when deciding which anti-obesity medication to prescribe, if at all?

- Very important
- Fairly important

- Not very important
- Not at all important
- Don’t know

A7. What do you believe are the main barriers to prescribing currently available anti-obesity medications, if any?

- Cost of treatments
- Side effects of treatment
- Patient adherence
- Lack of efficacious treatment
- Ease of access to effective methods
- Lack of awareness of anti-obesity medications
- Lack of psychological support
- Patient mindset towards prescription treatment
- Patient lack of trust in medication
- Mode of administration
- Lack of personal experience with anti-obesity medications
- Other
- There are no barriers
- Don’t know

A8. How involved are patients in making decisions about their anti-obesity medication, if at all?

- Very involved
- Fairly involved
- Not very involved
- Not at all involved
- Don’t know

A9. What anti-obesity medication(s), if any, have you prescribed for weight loss in the past 6 months?

- Saxenda (liraglutide 3mg)
- Xenical/ Alli (orlistat)
- Wegovy (semaglutide)
- Qysmnia (phentermine)
- Tenuate Retard (amfepramone)
- Mysimba (naltrexone/bupropion)
- IMCIVREE (setmelanotide)
- Mounjaro (tirzepatide)
- Trulicity (dulaglutide)
- I have not prescribed any anti-obesity medications for weight loss in the past 6 months
- Glucagon-like peptide-1 (GLP-1)
- Ozempic
- Topamax (topiramate)
- Glucophage (metformin)
- Plenity
- Farxiga and Forxiga (dapagliflozin)
- Rybelsus
- Sodium-glucose co-transporter-2 (SGLT2)

- Thyroxine
- Other
- Don’t know

SECTION B: PROFILE REVIEW

B1. How likely or unlikely would you be to prescribe product X?
- Very likely
- Fairly likely
- Neither likely nor unlikely
- Not very likely
- Not at all likely
- Don’t know

B2. What do you believe are the benefits, if any, of product X for patients living with obesity or overweight with a weight-related co-morbidity?
- Weight loss efficacy
- Once weekly dosing
- Cardiovascular risk factor improvements
- Appetite suppression
- Tolerable side effects
- Duration of treatment
- Injectable
- Another benefit
- No benefit
- Don’t know

B3. What are your main concerns, if any, of product X?
- Severity of side effects
- Injectable
- Duration of treatment
- No concerns
- Time taken to become effective
- Cost
- Appetite suppression
- Dosing
- Reimbursement/insurance coverage
- Don’t know
- Long-term safety data
- Guidelines
- No change in eating habits/ work on weight
- Patients rely too much on medication
- Accessibility
- Efficacy
- Wrong message to society
- Compliance
- Gastrointestinal side effects

- Lack of clinical data
- Regaining weight when stopping
- Cost benefit ratio
- Another concern

B4. To what extent, if at all, would the availability of product X overcome existing barriers in the current treatment landscape for weight loss?

- A great extent
- To some extent
- Hardly
- Not at all
- Don’t know

B5. How likely or unlikely would you be to switch your patients who are currently taking anti-obesity medications to product X?

- Very likely
- Fairly likely
- Neither likely nor unlikely
- Fairly unlikely
- Very unlikely
- Don’t know

B6. Which of the following, if any, would encourage you to recommend product X?

- Guidelines from health organisations/ medical societies in my country
- Recommendation from health authority/ HTA body in my country
- Failed on previous medication
- More information on product X
- Patient request
- Peers/colleagues recommending the product to their patients
- Sales rep visit on product X
- None of the above
- Don’t know

B7. Which of the following patient types, if any, would you recommend product X to?

- Patients with a BMI over 30
- Type 2 diabetes patients
- Patients who have failed to lose weight through diet and exercise only
- Patients living with overweight with weight related risk factors
- Patients using anti-obesity medication with more side effects
- Patients using less effective anti-obesity medication
- Patients on the waiting list for bariatric surgery
- Patients trying to lose weight
- None of the above
- Don’t know

B8. Which, if any, of the products you have just seen would you prefer to prescribe to your patients who are living with obesity or overweight?

- Product X
- Product Z

- Product Y
- Product A (US only)
- Product B (US only)
- No preference
- Don’t know

B9. You previously stated that you would prefer to prescribe **[product chosen by surveyed HCP from profiles shown]**, why is that?

- Weight loss efficacy
- Frequency of administration
- Mode of action
- Additional benefits
- Safety profile
- Method of administration
- Dosing
- Indication
- Another benefit
- Don’t know

B10. Given the choice, which of the products do you think most of your patients living with obesity or overweight would prefer to receive?

- Product X
- Product Z
- Product Y
- Product A (US only)
- Product B (US only)
- I believe my patients won’t have a preference
- Don’t know

FIGURE S1 Current methods of weight loss (A) among PwO and (B) prescribed/recommended by HCPs in the past 6 months.

A

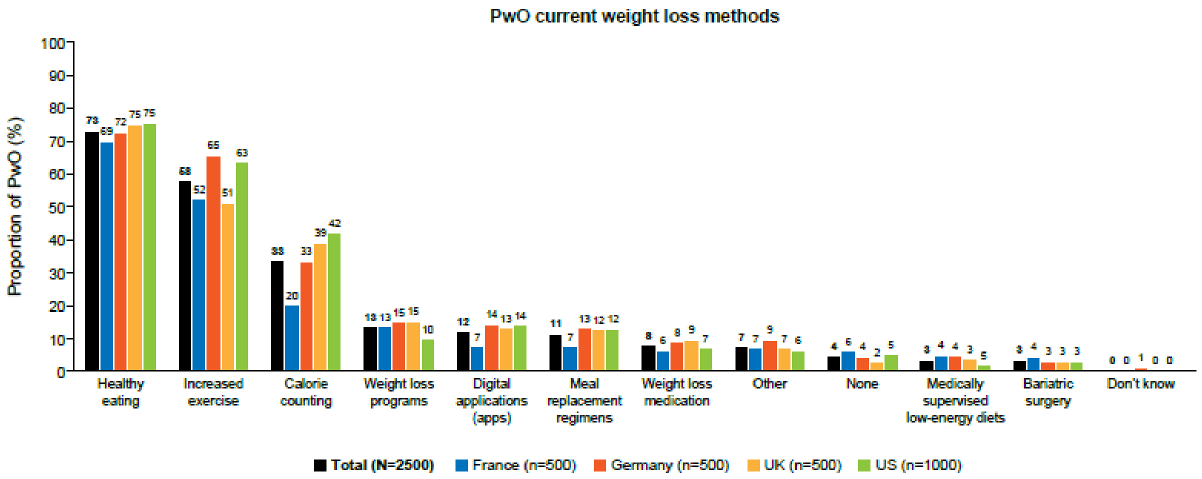

B

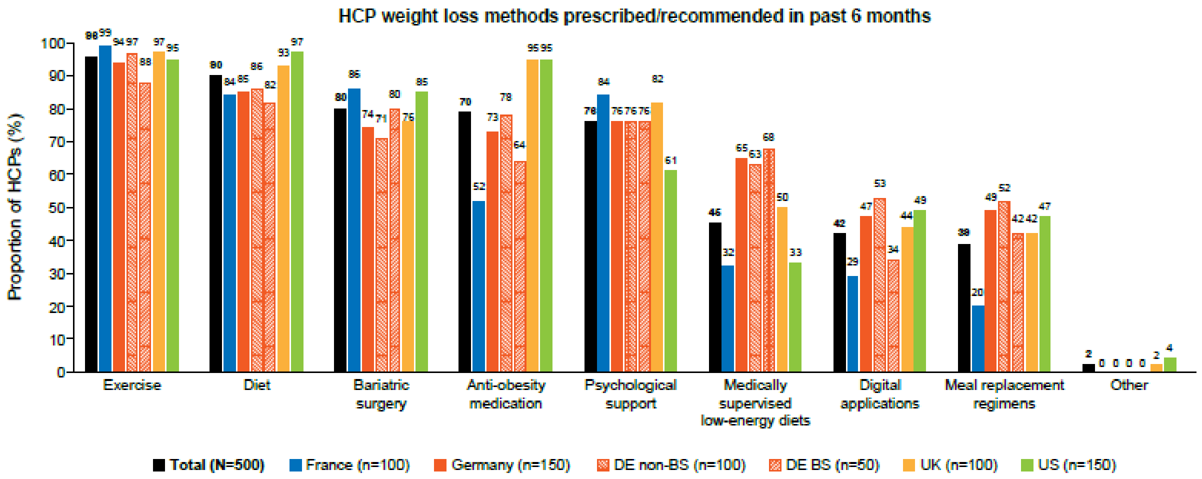

Abbreviations: BS, bariatric surgeon; DE, Germany; HCP, healthcare professional; PwO, people with obesity.
